# Nanotechnology’s advancement in diabetes mellitus regenerative medicine

**DOI:** 10.1097/MS9.0000000000000490

**Published:** 2023-04-05

**Authors:** Ilham Ben Amor, Deepak Chandran, Ruhul Amin, Talha B. Emran

**Affiliations:** a Department of Process Engineering and Petrochemical, Faculty of Technology, University of El Oued, El Oued, Algeria; b Laboratory of Biotechnology Biomaterials and Condensed Materials, Faculty of Technology, University of El Oued, El Oued, Algeria; c Department of Veterinary Sciences and Animal Husbandry, Amrita School of Agricultural Sciences, Amrita Vishwa Vidyapeetham University, Coimbatore, Tamil Nadu, India; d Faculty of Pharmaceutical Science, Assam Down Town University, Guwahati, Assam, India; e Department of Pharmacy, BGC Trust University Bangladesh, Chittagong, Bangladesh; f Department of Pharmacy, Faculty of Allied Health Sciences, Daffodil International University, Dhaka, Bangladesh

*Dear Editor*,

Diabetes is a severe global public health issue that affects individuals everywhere. Diabetes has a serious, long-term side effect called diabetic retinopathy. The primary cause of vision loss, diabetic retinopathy, is often present in patients in variable degrees. Examples of traditional treatments include laser therapy, antiangiogenesis, vitrectomy, and steroid hormones. These methods, however, cannot treat retinal ischemia and underlying neurodegenerative disease. As a result, there is no permanent solution to the diabetic retinopathy issue; rather, it has to be handled by promoting retinal tissue regeneration.

The rapid development of nanotechnology in recent years has improved the accuracy and efficacy of retinal tissue regeneration. The main advantage of nano-stents for the regeneration of retinal tissue is their chemical attributes that make it simpler to promote cell adhesion and increase biological effectiveness^1^.

Recently, the utilization of newly created conductive hydrogels and three-dimensional printing technology has helped the development of functional tissue engineering. Self-assembled nanopeptides could also mimic the environment of the extracellular matrix for the regeneration of nerve tissue, and with carbon nanotubes, nerve tissue may also be repaired. According to Koutsopoulos and colleagues, conductive carbon nanotubes can mimic the myelin sheath’s conductive function, enhance axon growth and regeneration, and encourage Schwann cell adhesion, migration, and proliferation^2^.

Diabetic foot is one of the most serious and costly long-term effects of diabetes. It is the primary cause of trauma-free diabetic amputations. A stem cell transplant improves the angiogenesis and wound healing of diabetic foot ulcers and lessens ischemia in the lower extremities^3^. Human umbilical vein endothelial cells and skin fibroblasts were grown by Mohandas and colleagues on chitosan-hyaluronic acid sponges and composites of nanofibril proteins. They discovered that the nanomaterial, when used as a trigger in the diabetic tissue engineering process of skin regeneration, may improve cell survival and adhesion as well as the potential for angiogenesis in healed wounds. Hyaluronic acid, chitosan, and nano-Ag sponges that are antibacterial and sterile nano dressings for diabetic foot ulcers have all been studied. These studies have discovered that the toxicity of a substance depends on its nanosilver content. The ideal concentration can be more beneficial to wound healing and have a significant antibacterial effect on wound tissue regeneration.

Significant myocardial necrosis may occur as a result of diabetic cardiomyopathy, also known as cardiac microangiopathy and myocardial metabolic disease. Reduced cardiac contractile cells due to altered levels of a particular cardiomyocyte protein and/or posttranslational alterations, which can cause arrhythmia, heart failure, cardiogenic shock, and sudden death, are the disorder’s major causes. Myocardial regeneration and repair can help treat the problem in some cases. However, the low survival rate seriously impairs the efficacy of stem cell implantation. In addition, a brand-new spherical nanoadhesive for myocardial regeneration was discovered by Hosoyama and colleagues. When rats’ cardiomyocytes are electrically stimulated, this nano-plaster can boost connexion expression and the density of neovascularization, assisting in the restoration of heart function^4^.

Methacrylate gelatin and Navaei gold nanorods created the cardiomyocyte regeneration matrix. They discovered that cardiac-specific markers like actin and connexin were expressed more often and that cell adhesion had increased^5^. Also, they discovered that cell activity, metabolic activity, and function were all significantly higher than those of a typical polyacrylic acid medium. As a consequence of the development and use of more unique nanomaterials, future advancements in diabetic regeneration medicine will undoubtedly be more significant, making them a key auxiliary technology of the area.

Nanotechnology has proven to have a crucial function as a catalyst in the repair of diabetic tissues and organs. Good histocompatibility of different nanoparticles lessens the transplant rejection of allogeneic cell tissue, which in turn reduces the need for lifelong immunosuppressants and offers patients with greater convenience. Tissue and organ transplantation, as well as pluripotent stem cell regeneration, can be monitored more sensitively and effectively with the help of nanotechnology’s monitoring and imaging tools. In the meanwhile, more research into the toxicity, safety, and side effects of these nanomaterials is necessary to minimize any potential harm to the body. In conclusion, nanotechnology has greatly aided the progress of regenerative medicine in the field of diabetes. It is inevitable that further research into and application of novel nanomaterials will lead to major advances in diabetic regenerative medicine, eventually making nanomaterials a vital auxiliary technology in this field.

## Ethical approval

None.

## Consent

All authors read the manuscript and agree for publications.

## Sources of funding

None.

## Author contribution

I.B.A. and D.C.: conceptualization, data curation, writing – original draft preparation, writing – reviewing and editing. R.A. and T.B.E.: writing – reviewing and editing, visualization, supervision.

## Conflicts of interest disclosure

The authors declare that they have no financial conflict of interest with regard to the content of this report.

## Research registration unique identifying number (UIN)

Not applicable.

## Guarantor

Talha B. Emran.

## Data availability statement

All data availabe in this manuscript.
